# COMUNET: a tool to explore and visualize intercellular communication

**DOI:** 10.1093/bioinformatics/btaa482

**Published:** 2020-05-12

**Authors:** Maria Solovey, Antonio Scialdone

**Affiliations:** b1 Institute of Computational Biology, Helmholtz Zentrum München – German Research Center for Environmental Health, Neuherberg 85764, Germany; b2 Institute of Epigenetics and Stem Cells, Helmholtz Zentrum München – German Research Center for Environmental Health, München 81377, Germany; b3 Institute of Functional Epigenetics, Helmholtz Zentrum München – German Research Center for Environmental Health, Neuherberg 85764, Germany

## Abstract

**Motivation:**

Intercellular communication plays an essential role in multicellular organisms and several algorithms to analyze it from single-cell transcriptional data have been recently published, but the results are often hard to visualize and interpret.

**Results:**

We developed Cell cOmmunication exploration with MUltiplex NETworks (COMUNET), a tool that streamlines the interpretation of the results from cell–cell communication analyses. COMUNET uses multiplex networks to represent and cluster all potential communication patterns between cell types. The algorithm also enables the search for specific patterns of communication and can perform comparative analysis between two biological conditions. To exemplify its use, here we apply COMUNET to investigate cell communication patterns in single-cell transcriptomic datasets from mouse embryos and from an acute myeloid leukemia patient at diagnosis and after treatment.

**Availability and implementation:**

Our algorithm is implemented in an R package available from https://github.com/ScialdoneLab/COMUNET, along with all the code to perform the analyses reported here.

**Supplementary information:**

Supplementary data are available at *Bioinformatics* online.

## 1 Introduction

Single-cell RNA sequencing (scRNA-seq) has been extensively used in the past few years to analyze intercellular communication in tissues (see e.g. Bonnardel *et al.*, 2019; Camp *et al.*, 2017; Caruso *et al.*, 2019; Cohen *et al.*, 2018; Halpern *et al.*, 2018; Kumar *et al.*, 2018; Puram *et al.*, 2017; Schiebinger *et al.*, 2019; Sheikh *et al.*, 2019; Skelly *et al.*, 2018; Vento-Tormo *et al.*, 2018; Wang, 2020; Zepp *et al.*, 2017; Zhou *et al.*, 2017). Several algorithms to perform these analyses have been published (for instance, Boisset *et al.*, 2018; Caruso *et al.*, 2019; Efremova *et al.*, 2020; Ramilowski *et al.*, 2015; Rieckmann *et al.*, 2017; Vento-Tormo *et al.*, 2018; Wang, 2020) and they all start from a database of interacting molecular partners (e.g. ligand and receptor pairs) to infer, from their expression patterns, a list of potential communication pathways between cell types.

While the results of these analyses can reveal important insights into the functioning of complex tissues composed of many different cell types, their interpretation can still be challenging with the existing algorithms.

For example, the standard visualization strategies based on graphs or heatmaps, are often busy, poorly interpretable and can hinder data-driven hypothesis generation. Given the sheer number of potentially communicating cell types and interacting partners, it can become challenging to identify all the molecules that could mediate the communication between a given set of cell types. Moreover, to understand the biological relevance of specific interacting molecular partners and the cellular communication they generate, it is desirable to be able to quantitatively compare cellular communication across different datasets.

Here, we present Cell cOmmunication exploration with MUltiplex NETworks (COMUNET), a new tool to visualize and interpret cell–cell communication that is based on multiplex networks and addresses all the challenges mentioned above. More specifically, COMUNET allows (i) unsupervised clustering of interacting partners (e.g. ligand–receptor pairs), (ii) search for specific patterns of communication and (iii) comparison between two biological conditions, aiding the interpretability of the results and the identification of promising candidate molecules to follow-up on. In this article, we show how COMUNET can perform these tasks on two scRNA-seq datasets from mouse embryos and from the bone marrow of an acute myeloid leukemia patient. COMUNET can be easily installed and run, since it is available as an R package from https://github.com/ScialdoneLab/COMUNET.

## 2 Materials and methods

### 2.1 Multiplex networks

**Fig. 1.**
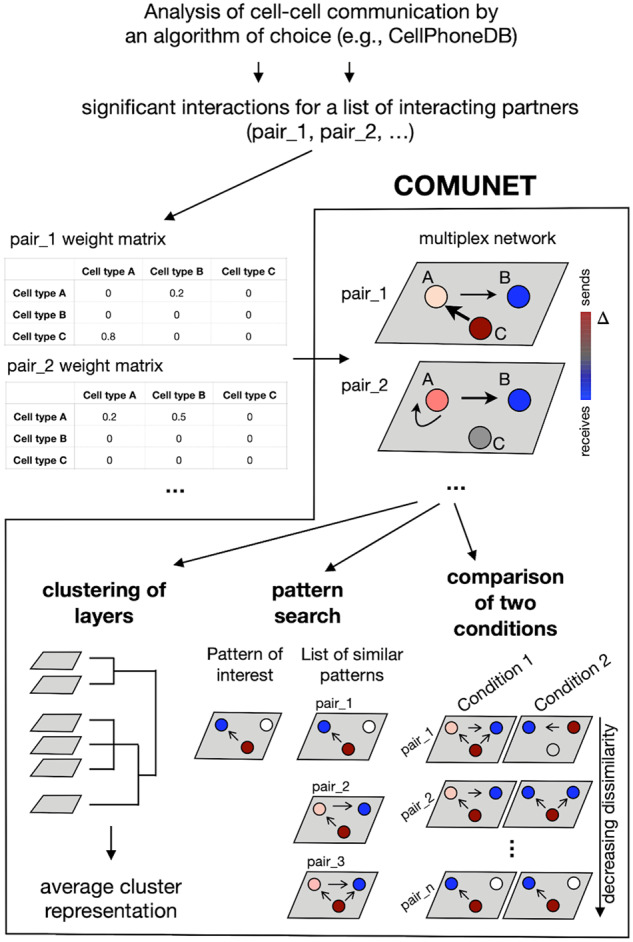
Schematic representation of COMUNET workflow. Once the weight matrices for a set of interacting partners (e.g. ligands–receptor pairs) are estimated with an algorithm of choice (e.g. CellPhoneDB), COMUNET represents them as layers in a multiplex network, where nodes are cell types (indicated with A, B and C). Each node is colored based on the difference between the weighted in- and out-degree (indicated with Δ), in such a way that, in the case of ligands/receptors, the nodes that preferentially send signals are red, whereas the nodes that preferentially receive signals are blue. Next, COMUNET calculates pairwise dissimilarities between layers in the multiplex network and can perform: clustering of layers, to reveal interacting partners sharing similar communication patterns; search of interacting partners showing a specific communication pattern; comparison of communication patterns between two biological conditions.

As a first step, COMUNET uses a multiplex network to represent all significant interacting partners (consisting of, e.g. pairs of ligands and receptors) that can mediate cell–cell communication identified by an algorithm of choice. For the sake of definiteness, in the examples below we use CellPhoneDB (Efremova *et al.*, 2020) to find significant interacting partners. However, COMUNET can work with any algorithm [e.g. ImmProt (Rieckmann *et al.*, 2017) or any of the other algorithms cited in Section 1] that provides a quantification of the strength/statistical significance of the communication between cell types mediated by a given pair of interacting partners. This information can be represented in the form of a matrix (see Fig. 1), which from now on we will refer to as the weight matrix. The input of COMUNET is a set of weight matrices, one for each pair of interacting partners (Fig. 1).

Each weight matrix is interpreted as an adjacency matrix of a directed weighted graph, where the entries are the edge weights. For each node (representing cell types), the difference between the weighted in- and out-degree, Δ, is calculated and encoded in the color of the node.

If the pair of interacting partners is a ligand–receptor pair, a positive value of Δ indicates that the node (cell type) is mostly communicating by producing the ligand (‘sending’ node); conversely, a negative Δ marks nodes that communicate mainly by receiving signals through the receptor (‘receiving node’). The arrows of the graph start at the sending nodes (which express the ligand) and point to the receiving nodes (which express the receptor), whereas the thickness of an edge indicates the edge weight.

In case, no directionality is specified for the pair of interacting partners P1 and P2 (i.e. as for adhesion molecules), the arrows start at the node expressing partner P1 and points to the node expressing partner P2. The graphs built from the matrices are then stacked together as layers of a multiplex network (Fig. 1).

### 2.2 Dissimilarity measure

Once the pairs of interacting partners are represented as layers in a multiplex network, COMUNET calculates pairwise dissimilarities between them.

Given two layers α and β, their dissimilarity $d^{\alpha ,\beta }$ is defined as:

$$d^{\alpha ,\beta }=\frac{\sum_{i,j=1}^{N}s_{i,j}^{\alpha ,\beta }}{\left|E^{\alpha }\cup E^{\beta }\right|}$$

with

$$s_{i,j}^{\alpha ,\beta }=\{\begin{array}{cc} \frac{\left|w_{i,j}^{\alpha }-w_{i,j}^{\beta }\right|}{w_{i,j}^{\alpha }+w_{i,j}^{\beta }} & \text{for}\;w_{i,j}^{\alpha }\;w_{i,j}^{\beta }\;\neq 0\\ 0 & \text{otherwise} \end{array}$$

where $w_{i,\;j}^{\alpha }$ is the weight of the directed edge between nodes i and j in the layer α, N is the total number of nodes, $E^{\alpha }$ is the set of edges in the layer α, $\left|E^{\alpha }\cup E^{\beta }\right|$ is the cardinality of the union of all edges in layers α and β. This definition of dissimilarity can be seen as a modified version of the Jaccard similarity index (Jaccard, 1912) between the sets of edges in the two layers that also takes into account the weights and directionality of the edges.

### 2.3 Clustering of interacting partners

In this step, COMUNET identifies pairs of interacting partners with similar patterns of intercellular communication and organizes them into clusters (Fig. 1).

**Fig. 2.**
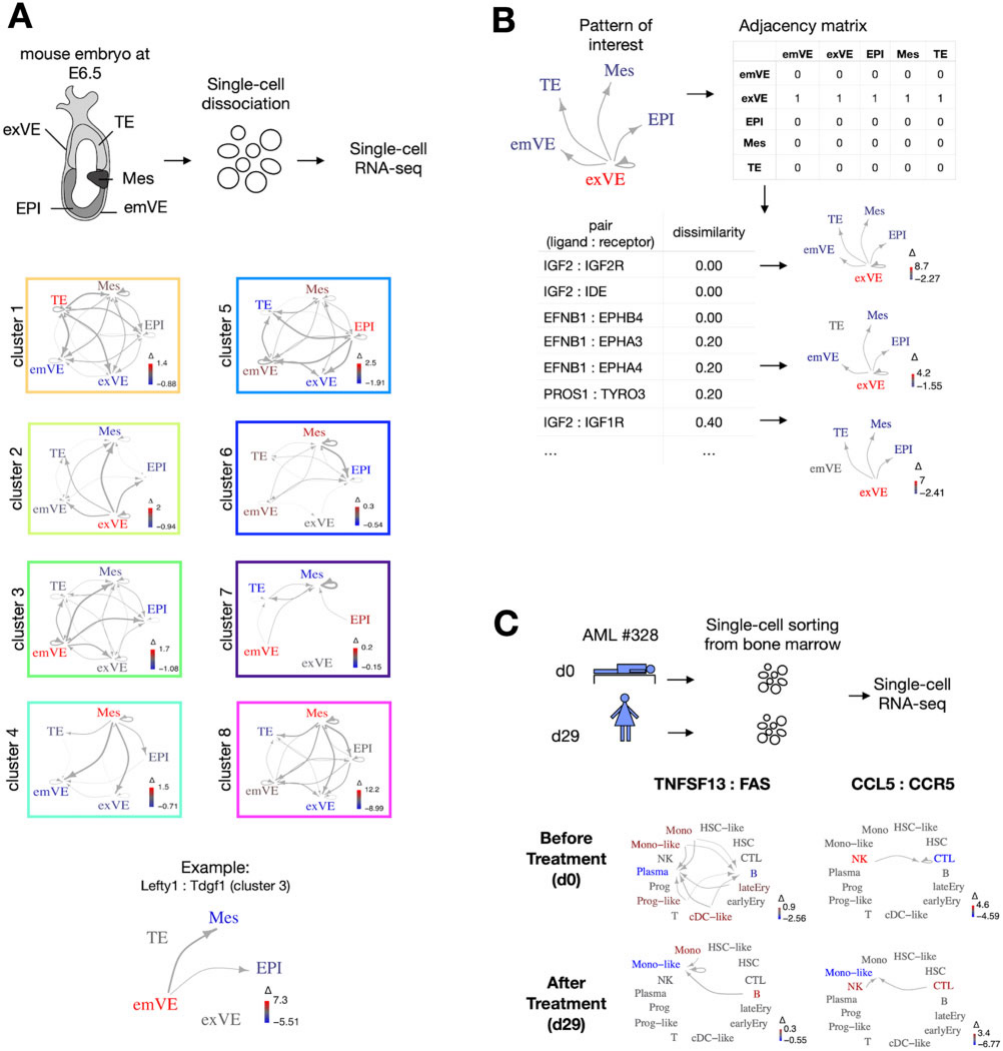
Application of COMUNET to datasets from mouse embryos and cancer. (**A**) We used COMUNET to identify clusters of interacting partners in published scRNA-seq data from E6.5 mouse embryo. The dataset includes five cell types: extraembryonic visceral endoderm (exVE), trophectoderm (TE), mesoderm (Mes), embryonic visceral endoderm (emVE) and epiblast (EPI). Eight clusters of interacting partners were identified, each corresponding to a specific communication pattern, whose average representation is depicted in the squares. In particular, Cluster 3 represents communication from the emVE to EPI and Mes, Lefty1:Tdgf1 being a representative ligand:receptor pair included in this cluster. **(B**) As an example of pattern search, using the same scRNA-seq data of panel A, we searched for the interacting partners showing the pattern of communication depicted in the top left: i.e. a signal originating from the extraembryonic visceral endoderm and received by all other embryonic tissues, which corresponds to the adjacency matrix shown in the top right. COMUNET returned a list of interacting partners sorted by increasing dissimilarity with the specified pattern (bottom left). The bottom right panel illustrates the graphs corresponding to some selected pairs of interacting partners. (**C**) We applied COMUNET to a published scRNA-seq dataset from the bone marrow of an AML patient at diagnosis (d0) and after treatment (d29) to find differences in communication patterns between the two time points. TNFSF13:FAS and CCL5:CCR5 are examples of interacting pairs with a dramatic change of communication patterns between d0 and d29.

This is done by performing hierarchical clustering of the dissimilarity matrix of layers (by default, the ‘hclust’ R function with the ‘average’ agglomeration method is used) and the results can be visualized as a heatmap or a UMAP plot (McInnes *et al.*, 2018). The number of clusters is estimated using the ‘cutreeHybrid’ R function [package dynamicTreeCut, version 1.63-1(Langfelder *et al.*, 2008)] with ‘deepSplit’ equal to 0 and default ‘minClusterSize’ equal to 6. For each cluster, a graph that represents the ‘average’ pattern is built by averaging the adjacency matrices and the Δ (see Section 2.1) of the nodes of all the graphs in the cluster (Figs 1 and 2A).

### 2.4 Cell–cell communication pattern search

For a more supervised analysis, it is useful to retrieve all pairs of interacting partners that show a specific communication pattern. To this aim, our algorithm classifies the pairs of interacting partners based on their similarity to a user-specified intercellular communication pattern.

A pattern of interest is defined with a binary adjacency matrix, 1 indicating the presence of an edge and 0 the absence. The adjacency matrices of the layers are also binarized based on the presence/absence of the edges and then the dissimilarities with the user-specified adjacency matrix are calculated (see Section 2.2). The output is a list of pairs of interacting partners sorted by increasing dissimilarity with the user-specified pattern (Figs 1 and 2B, Supplementary Table S2).

### 2.5 Comparative analysis

Given the results of a cell–cell communication analysis of two datasets including the same cell types, COMUNET can estimate the differences in the intercellular communication patterns between corresponding interacting partners in the two datasets. First, the results are represented as multiplex networks, as described above; then, pairwise dissimilarities between the shared layers (e.g. layers representing the same pairs of interacting partners) of the two datasets are calculated (see Section 2.2). The pairs of interacting partners are then sorted by decreasing dissimilarity, i.e. from those that change the most across the two datasets to those that remain unaltered (Figs 1 and 2C, Supplementary Table S3).

## 3 Results

Below, we apply COMUNET to scRNA-seq datasets from two different publications to show how the clustering, the pattern search and the comparative analysis can help extract biologically relevant information from a cell–cell communication analysis performed with CellPhoneDB (Efremova *et al.*, 2020; Vento-Tormo *et al.*, 2018). In the first example, we used COMUNET to characterize the cellular communication patterns in a mouse embryo dataset, and in the second example, we found the changes in cellular communication in the bone marrow of a leukemia patient between time of diagnosis and after treatment.

### 3.1 E6.5 mouse embryo

We took scRNA-seq data from a mouse embryo at the E6.5 stage from Nowotschin *et al.* (2019) (Fig. 2A, Supplementary Fig. S1). Around this stage gastrulation is starting, with signaling between cell types that begin to define where and when the first organs will arise (Tam and Loebel, 2007). As an example, the external layer of cells called visceral endoderm, in addition to being responsible for nutrient uptake and transport, determines the direction of the anterior–posterior axis and the region of epiblast where mesodermal cells will differentiate, through mechanisms and signaling pathways that are still not fully understood (Stower and Srinivas, 2018). On this dataset, including five different cell types, we ran CellPhoneDB and then the clustering algorithm of COMUNET, which finds eight clusters (see Fig. 2A, Supplementary Table S1). These clusters classify the interacting partners according to the pattern of communication they might generate between the different cell types and offer an overview of the main communication patterns present in the data.

As an example, if we consider the ligands/receptor pairs in Cluster 3, they are responsible for signaling that originates preferentially from the embryonic visceral endoderm (emVE) and is directed to other cell types (Fig. 2A, Supplementary Fig. S1). Indeed, this cluster includes LEFTY1-TDGF1 (Fig. 2A;Supplementary Fig. S1 and Supplementary Table S1), which are part of the nodal signaling that regulates expression of mesodermal genes in the epiblast (Tam and Loebel, 2007).

In addition to this unsupervised analysis, all interacting partners having a specific communication pattern can be identified. For example, in Figure 2B and Supplementary Table S2, we show how a list of all interacting partners that might be responsible for signaling from the extraembryonic visceral endoderm to all the other cell types can be easily obtained. At the top, we found Igf2, which is known to promote embryonic growth (DeChiara *et al.*, 1991), and its receptor Igf2r, which attenuates Igf2 signaling (Brown *et al.*, 2009).

### 3.2 Human acute myeloid leukemia dataset

There is growing evidence that communication between different cell types in a tissue is altered in diseases like cancer. For instance, in healthy bone marrow, hematopoietic stem cells stay in close contact with niche cells, including endothelial cells, megakaryocytes and adipocytes (Asada *et al.*, 2017; Behrmann *et al.*, 2018), which regulate their proliferation state (Ambrosi *et al.*, 2017; Bruns *et al.*, 2014; Itkin *et al.*, 2016). In hematological malignancies as well as solid cancers, the normal control mechanisms executed by the niche cells are disrupted, such that a novel tumor-specific communicational landscape dominates the tissue. Tumor cells can actively recruit the niche cells from their environment (e.g. fibroblasts, mesenchymal stem cells, T cells or macrophages) and receive growth factors secreted by the niche, to evade from the immune response or to metastasize (Behrmann *et al.*, 2018; Yuan *et al.*, 2016). To investigate the alteration of communication patterns in cancer, it is crucial to have a tool that can perform the communication comparison between conditions, such as before and after treatment.

In this example, we used the data from (van Galen *et al.*, 2019) to show how COMUNET can find differences in communication patterns between two datasets. This publication includes a scRNA-seq data of a bone marrow sample of a 74 years old newly diagnosed female patient with acute myeloid leukemia (AML) (#328) at diagnosis (day 0) and after Azacitidine-Venetoclax-C1D27 treatment (day 29). At the two timepoints, the bone marrow of this patient contains both normal hematopoietic cells, as well as tumor cells (HSC-like, progenitor-like, monocyte-like and cDC-like) (Supplementary Fig. S2). We compared the communication patterns in the two samples and revealed that six pairs of interacting partners dramatically change their pattern of communication upon treatment. Among those we found TNFSF13: FAS and CCL5: CCR5, two pairs of ligands/receptors that are known to play a role in AML and other cancers (Aldinucci and Colombatti, 2014; Brenner *et al.*, 2016; Chapellier *et al.*, 2019; Gmeiner *et al.*, 2015; Mollica Poeta *et al.*, 2019) (Fig. 2C, Supplementary Fig. S2 and Table S3). In AML, TNFSF13, secreted by normal myeloid cells, supports proliferation and apoptosis resistance of tumor cells (Chapellier *et al.*, 2019). In solid cancers, inflammatory chemokines such as CCL5 together with their receptors have been shown to attract neutrophils and monocytes at the tumor site and reprogram them, leading to altered immune response (Mollica Poeta *et al.*, 2019). This signaling might be relevant for the immune response and survival of a subset of tumor cells in this patient after treatment.

## 4 Discussion

In this article, we introduced COMUNET, a powerful tool for visualization and analysis of intercellular communication. Starting from the output generated by any algorithm that estimates the likelihood or strength of cellular communication, COMUNET represents the pattern of cell communication mediated by each pair of interacting molecules as layers in a multiplex network and calculates the dissimilarity between them. Based on this dissimilarity, COMUNET clusters the pairs of interacting partners based on the pattern of intercellular communication that they can mediate. This facilitates the visualization of the results and gives an overview of the cell types that are involved in the communication, in addition to the molecules that could be responsible for it. Furthermore, COMUNET can prioritize the molecules that are more likely to mediate the communication between a set of specific cell types.

We showed how COMUNET can perform these two tasks in a dataset from mouse embryos, where we classified the communication patterns in eight different clusters and we were able to easily retrieve a list of potential mediators for signaling originating from extraembryonic visceral endoderm.

A comparison of cellular communication between two different conditions is often necessary to understand the role of signaling molecules. COMUNET can perform such a comparison to identify the changes in communication and the molecules that are responsible for them. We showed this in a dataset from an AML bone marrow before and after treatment and we were able to single out molecules that were previously linked with important processes like apoptosis resistance and immune response in cancer.

These examples show how COMUNET significantly increases our ability to go beyond a simple descriptive study of cellular communication by helping to generate hypotheses on the role of communication and to identify candidate genes for validation.

Another feature of COMUNET is the possibility to use it on the output generated by any algorithm that quantifies intercellular communication. This is particularly important given the rapid pace at which new, refined algorithms for cellular communication are being developed and holds true irrespective of the kind of data used to infer communication, including, for instance, single-cell proteomics data (Labib and Kelley, 2020), which can be even more informative on cellular communication than RNA-seq.

## 5 Data and code availability

COMUNET is available as an R package from https://github.com/ScialdoneLab/COMUNET. In this GitHub repository, a comprehensive tutorial is provided with all the code and the data to reproduce the analyses shown in this article.

## Supplementary Material

btaa482_Supplementary_Data
