# Supplementary material for: COMUNET: a tool to explore and visualize intercellular communication
Source: Bioinformatics. 2020 May 12;36(15):4296–300. doi: 10.1093/bioinformatics/btaa482 (PMC7520036; doi:10.1093/bioinformatics/btaa482)
Supplement: btaa482_Supplementary_Data [file btaa482_supplementary_data.zip › btaa482-suppl_data/Suppl_Fig_1_2_legends_revised.pdf]

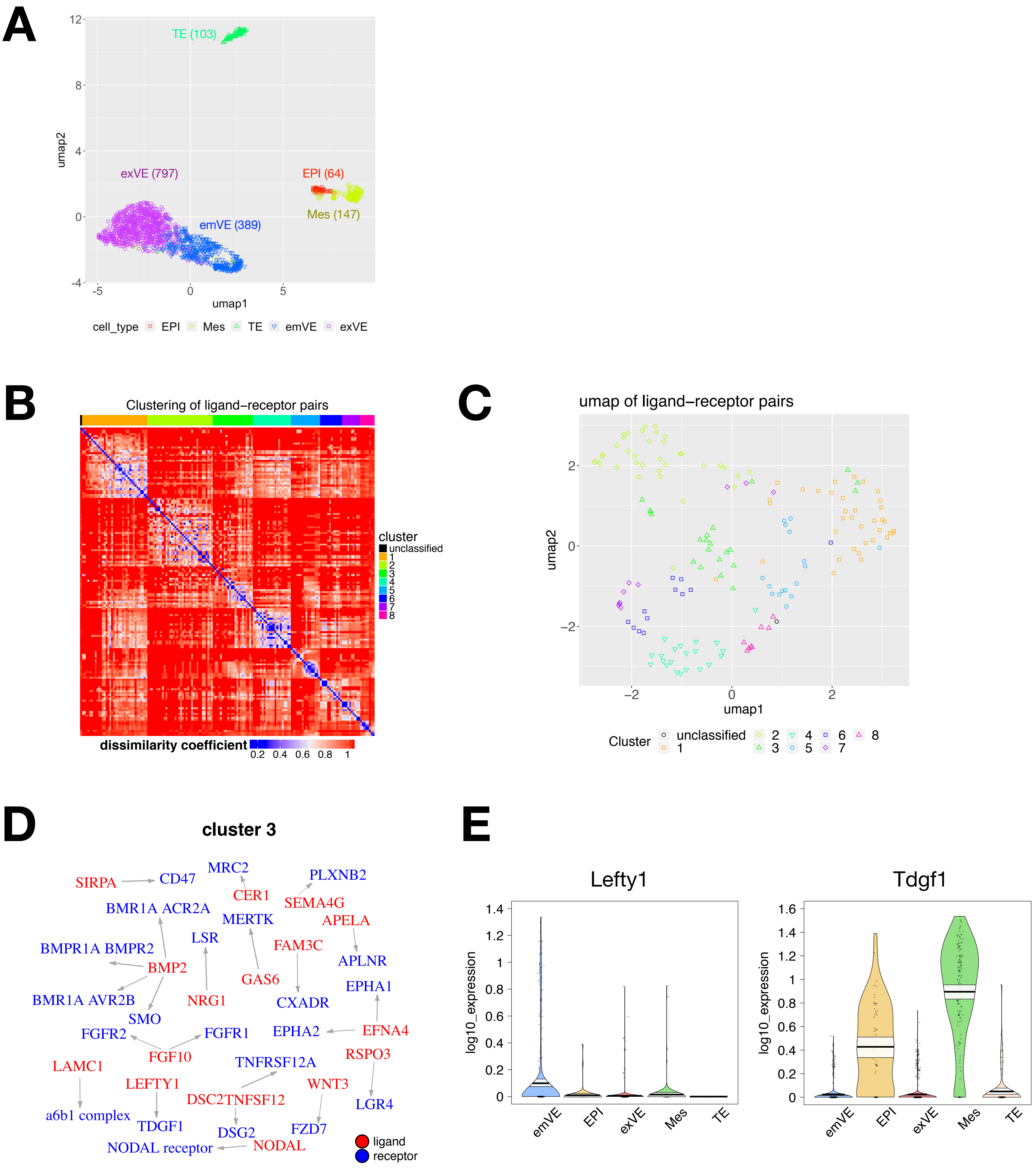
A
TE (103)
exVE (797)
EPI (64)
Mes (147)
emVE (389)
umap1
umap2
cell_type EPI Mes TE emVE exVE
B
Clustering of ligand−receptor pairs
cluster
unclassified
1
2
3
4
5
6
7
8
dissimilarity coefficient
0.2 0.4 0.6 0.8 1
C
umap of ligand−receptor pairs
umap1
umap2
Cluster unclassified 1 2 3 4 5 6 7 8
D
cluster 3
SIRPA
CD47
MRC2
PLXNB2
CER1
SEMA4G
BMR1A ACR2A
MERTK
APELA
LSR
BMPR1A BMPR2
FAM3C
APLNR
BMP2
GAS6
EPHA1
BMR1A AVR2B
NRG1
CXADR
SMO
FGFR2
FGFR1
EPHA2
EFNA4
FGF10
TNFRSF12A
RSPO3
LAMC1
LEFTY1
WNT3
DSC2TNFSF12
LGR4
a6b1 complex
TDGF1
DSG2
FZD7
NODAL receptor
NODAL
ligand
receptor
E
Lefty1
Tdgf1
log10_expression
emVE EPI exVE Mes TE

Supplemental Figure 1 - E6.5 mouse embryo data

A. UMAP of the E6.5 mouse embryo scRNA-seq data (supset to 1500 cells). Cell types are: embryonic visceral endoderm (emVE), epiblast (EPI), extraembryonic visceral endoderm (exVE), mesoderm (Mes), and trophectoderm (TE).
B. Heatmap showing the dissimilarity of interacting partners in the scRNA-seq data from E6.5 mouse embryo (supset to 1500 cells). Rows and columns are sorted by cluster number, the color represents dissimilarity between two pairs with 0 for identical pattern (blue), 1 for completely different pattern (red).
C. UMAP ofinteracting partners colored by cluster.
D. interacting partners in cluster 3. Ligands are represented in red, receptors in blue; arrows go from the ligand to the associated receptor.
E. Log10 expression levels of Lefty1 and Tdgf1.

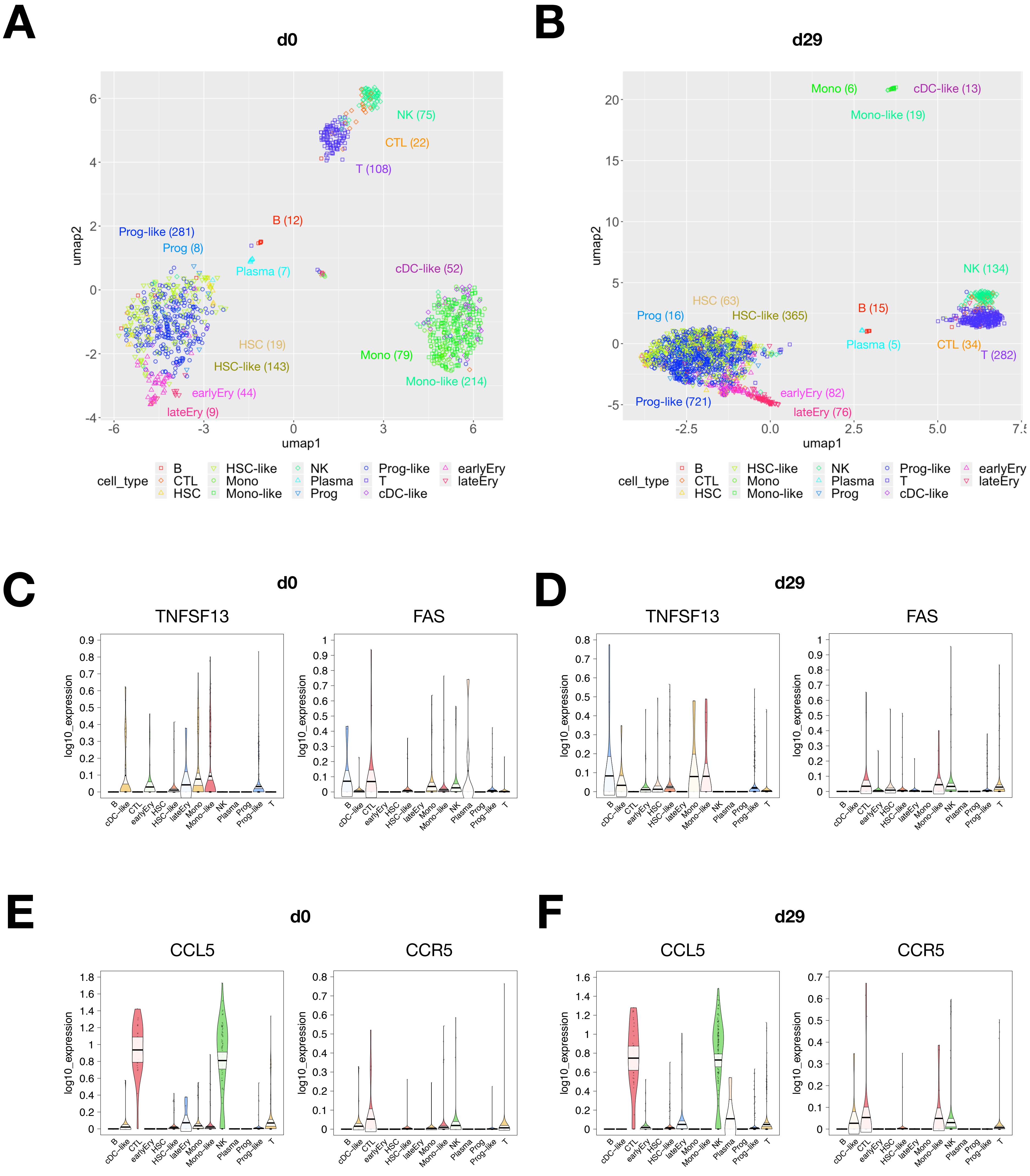
A
d0
NK (75)
CTL (22)
T (108)
B (12)
Prog-like (281)
Prog (8)
Plasma (7)
cDC-like (52)
HSC (19)
HSC-like (143)
Mono (79)
Mono-like (214)
earlyEry (44)
lateEry (9)
umap1
umap2
cell_type
B
CTL
HSC
HSC-like
Mono
Mono-like
NK
Plasma
Prog
Prog-like
T
cDC-like
earlyEry
lateEry
B
d29
Mono (6)
cDC-like (13)
Mono-like (19)
NK (134)
Prog (16)
HSC (63)
HSC-like (365)
B (15)
Plasma (5)
CTL (34)
T (282)
Prog-like (721)
earlyEry (82)
lateEry (76)
umap1
umap2
C
d0
TNFSF13
FAS
log10_expression
D
d29
TNFSF13
FAS
log10_expression
E
d0
CCL5
CCR5
log10_expression
F
d29
CCL5
CCR5
log10_expression
B
cDC-like
CTL
earlyEry
HSC
HSC-like
lateEry
Mono
Mono-like
NK
Plasma
Prog
Prog-like
T

Supplemental Figure 2 - AML patient data

A-B. UMAP of bone marrow of AML patient at d0 (panel A) and d29 (panel B). Number of cells in each population is represented in brackets.

C-D. Log10 expression levels of TNFSF13 and FAS at d0 (panel C) and d29 (panel D).

E-F. Log10 expression levels of CCL5 and CCR5 at d0 (panel E) and d29 (panel F).
